# Sequence-structure relations of pseudoknot RNA

**DOI:** 10.1186/1471-2105-10-S1-S39

**Published:** 2009-01-30

**Authors:** Fenix WD Huang, Linda YM Li, Christian M Reidys

**Affiliations:** 1 Center for Combinatorics, LPMC-TJKLC, Nankai University, Tianjin 300071, PR China

## Abstract

**Background:**

The analysis of sequence-structure relations of RNA is based on a specific notion and folding of RNA structure. The notion of coarse grained structure employed here is that of canonical RNA pseudoknot contact-structures with at most two mutually crossing bonds (3-noncrossing). These structures are folded by a novel, *ab initio *prediction algorithm, cross, capable of searching all 3-noncrossing RNA structures. The algorithm outputs the minimum free energy structure.

**Results:**

After giving some background on RNA pseudoknot structures and providing an outline of the folding algorithm being employed, we present in this paper various, statistical results on the mapping from RNA sequences into 3-noncrossing RNA pseudoknot structures. We study properties, like the fraction of pseudoknot structures, the dominant pseudoknot-shapes, neutral walks, neutral neighbors and local connectivity. We then put our results into context of molecular evolution of RNA.

**Conclusion:**

Our results imply that, in analogy to RNA secondary structures, 3-noncrossing pseudoknot RNA represents a molecular phenotype that is well suited for molecular and in particular neutral evolution. We can conclude that extended, percolating neutral networks of pseudoknot RNA exist.

## Background

Three decades ago, Michael Waterman pioneered the combinatorics and *ab initio *prediction of the at that time rather exotic ribunucleic acid (RNA) secondary structures [1-5]. The motivation for this work was coming from a fundamental dichotomy represented by RNA. On one hand RNA is described by its primary sequence, a linear string composed of the nucleotides **A**, **G**, **U **and **C**. The primary sequence embodies the genotypic legislative. On the other hand, RNA, being less structurally constrained than its chemical relative DNA, does fold into 3D-structures, representing the phenotypic executive. Therefore one molecule stands for both: geno- and phenotype.

Indeed, a vast variety of RNA activities was found: the discovery of catalytic RNAs, or ribozymes, in 1981 proved that RNA could catalyze reactions just as proteins. RNA can act also as a messenger between DNA and protein in the form of transfer RNA. The realization that RNA combines features of proteins with DNA led to the "RNA world" hypothesis for the origin of life. The idea was that DNA and the much more versatile proteins took over RNA's functions in the transition from the "RNA-world" to the "DNA/protein-world".

**Figure 1:**
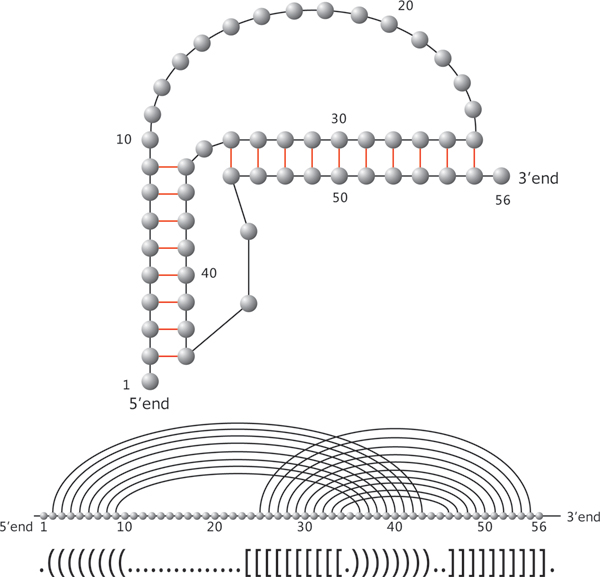
**RNA pseudoknot structures**. Three representations of the UTR-pseudoknot structure of the mouse hepatitis virus. First, the planar graph representation, second the diagram representation and finally the output produced by cross.

**Figure 2:**
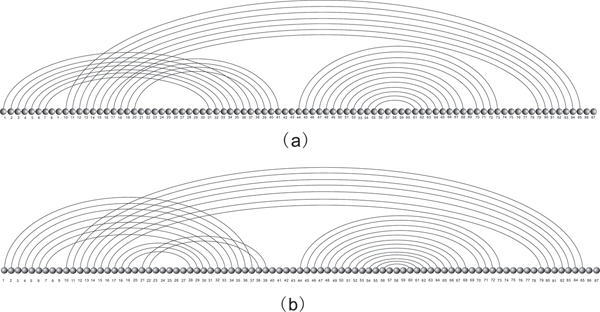
**HDV structure**. (a) Diagram representation of Hepatitis Delta Virus structure folded by our algorithm. (b) Diagram representation of natural Hepatitis Delta Virus.

Let us have a closer look at RNA phenotypes. RNA molecules form "helical" structures by folding, i.e. pairing their nucleotides and thereby lowering their minimum free energy (mfe). Originally, these bonds were subject to strict combinatorial constraints, for instance "noncrossing" in RNA secondary structures. For the latter, dynamic programming (DP) algorithms, predicting the minimum free energy configuration were given 1980 [5,6]. It is wellknown, however, that RNA structures are far more complex than secondary structures. One particularly prominent feature is the existence of cross-serial dependencies [7], that is crossing arcs or pseudoknots, see Figure 1, where we display the natural UTR-pseudoknot structure of the mouse hepatitis virus. Cross also folds into the natural structure given in Figure 1. In Figure 2 we present another RNA pseudoknot structure, the HDV-pseudoknot. We present here the structure as folded by cross and also its natural structure [8].

In fact, RNA pseudoknots are "everywhere". They occur in functional RNA, like for instance RNAseP [9] as well as ribosomal RNA [10]. They are conserved in the catalytic core of group I introns, in plant viral RNAs pseudoknots mimic tRNA structure and in *in vitro *RNA evolution [11], where experiments produced families of RNA structures with pseudoknot motifs, when binding HIV-1 reverse transcripts. Important mechanisms like ribosomal frame shifting [12] also involve pseudoknot interactions.

For prediction algorithms the implications of cross-serial dependencies are severe-they imply a higher level of formal language: context-sensitive. In general, on this level of formal languages it is not clear whether or not polynomial time *ab initio *folding algorithms exist. Indeed, Lyngsø *et al*. [13] showed that "reasonable" classes of RNA pseudoknots require exponential time algorithms. There exist however, polynomial time folding algorithms, capable of the energy based prediction of certain pseudoknots: Rivas *et al*. [14], Uemura *et al*. [15], Akutsu [16] and Lyngsø [13]. The output of these algorithms, however, remains somewhat "mysterious"-it is not clear which types of pseudoknots can be generated.

In analogy to the case of RNA secondary structures, the identification of key combinatorial properties of the output class offers deeper understanding. The combinatorial properties of RNA pseudoknot structures discussed in the following have indeed profound implications: first sequence-structure maps will generate exponentially many structures with neutral networks of exponential size. Second, the latter will come close to each other in sequence space, thereby allowing for efficient evolutionary search. None of these findings depend on the particular choice of loop-energies or the partition function [17]. Furthermore, without combinatorial specification, as it is the case for the above mentioned DP based pseudoknot folding algorithms [14], one arrives at an impossibly large configuration space.

For instance, the inductive generation of gap-matrices produces arbitrarily high number of mutually crossing arcs. The results in [18] prove, that the exponential growth rate of pseudoknot structures is *linear *in the crossing number. Accordingly, via gap-matrices, an uncontrollably large output class is being generated. Nevertheless, the DP-routine using pairs of gap-matrices cannot generate any 3-noncrossing *nonplanar *pseudoknot structure.

**Figure 3:**
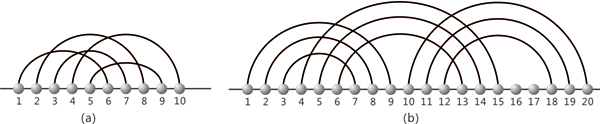
***k*-noncrossing diagrams**. We display a 4-noncrossing diagram with arc-length *λ *≥ 4 and stack-length *τ *≥ 1 (a) and a 3-noncrossing, *λ *≥ 4 and *τ *≥ 3 diagram (b).

**Figure 4:**
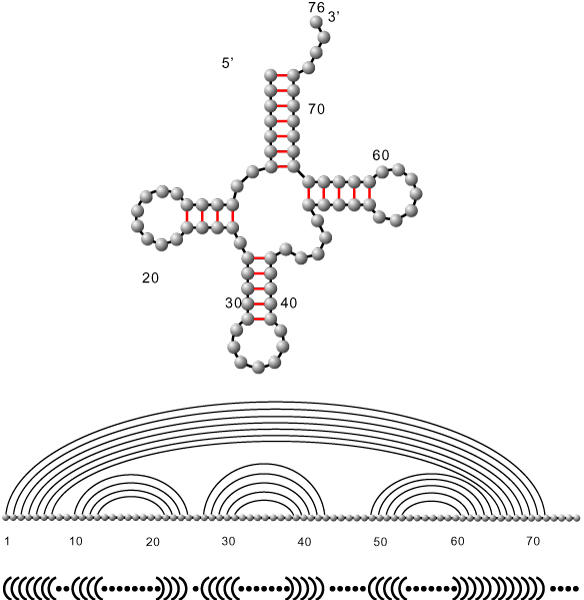
**RNA secondary structures: **Here we give three representations of the phenylalanine secondary (2-noncrossing) structure. First the outer-planar graph representation (top), second the diagram representation (middle) and finally the '.', '(' and ')' representation.

We will show that the notion of *k*-noncrossing diagrams [19] allows us to specify a suitable output-class for pseudoknot folding algorithms. Recall that a diagram is a graph over the vertex set [*n*] = {1, ..., *n*} with vertex degree less than or equal to one. It is represented by drawing the vertices in a horizontal line and its arcs (*i*, *j*), where *i *<*j*, in the upper half-plane. The vertices and arcs correspond to nucleotides and Watson-Crick (**A-U**, **G-C**) and (**U-G**) base pairs, respectively. A diagram is *k*-noncrossing if it contains at most *k *- 1 mutually crossing arcs. Diagrams have the following three key parameters: the maximum number of mutually crossing arcs, *k *- 1, the minimum arc-length, *λ*, and minimum stack-length, *τ*, The length of an arc (*i*, *j*) is *j *- *i *and a stack of length *τ *is a sequence of "parallel" arcs of the form

((*i*, *j*), (*i *+ 1, *j *- 1), ..., (*i *+ (*τ *- 1), *j *- (*τ *- 1))),

see Figure 3. We call an arc of length *λ *a *λ*-arc. Biophysical constraints on the base pairings imply that in all RNA structures *λ *is greater than or equal to four. We call diagrams with a minimum stack-length *τ*, *τ*-canonical and if *λ *≥ 4 we refer to diagrams as structures. To reiterate, in the simplest case we have 2-noncrossing RNA structures, i.e. the secondary structures in which no two arcs cross, see Figure 4. The noncrossing of arcs has far-reaching consequences. It implies that RNA secondary structures form a context free language and allow for the DP algorithms [20], predicting the loop-based mfe-secondary structure in *O*(*n*^3^)-time and *O*(*n*^2^)-space.

Let us now, having some background on RNA structures return to the RNA-world. Around 1990 Peter Schuster and his coworkers initiated a paradigm shift. They began to study evolutionary optimization and neutral evolution of RNA via the relation between RNA genotypes and phenotypes. The particular mapping from RNA sequences into RNA secondary structures was obtained by the algorithm *ViennaRNA *[21], an implementation of the folding routine [6,22], mentioned above. Two particularly prominent results of this line of work were the existence of neutral networks, i.e. vast, extended networks, composed of sequences folding into a given secondary structure [23] and the Intersection Theorem [23]. The latter guarantees for any two secondary structures the existence of at least one sequence which simultaneously satisfies all constraints imposed by their Watson-Crick and **G-U **base pairs. For the implication of the latter with respect to molecular switches, see [24]. It became evident that the "statistical" properties of this mapping played a central role in the molecular evolution of RNA.

But, there is more. Two discoveries suggested that RNA might not just be a stepping stone towards a DNA/protein world. They show that RNA plays an active role in vital cell processes. A large number of very small RNAs of about 22 nucleotides in length, called microRNAs (miRNAs), were discovered. They were found in organisms as diverse as the worm Caenorhabditis organs and humans, and their particular relationship to certain intermediates in RNA interference (RNAi). These findings have put RNA-in particular noncoding RNA-into the spotlight. In addition, RNA's conformational versatility and catalytic abilities have been identified in the context of protein synthesis and RNA splicing. More and more parallels between RNA and protein are currently being revealed [25].

Let us next briefly overview what we know about the combinatorics of our phenotypes, ultimatively allowing for the computation of biophysically relevant pseudoknot structures [26]. The key result comes from a seemingly unrelated field, the combinatorics of partitions. Chen *et al*. proved in a seminal paper [27] a bijection between walks in Weyl chambers and *k*-noncrossing partitions. This bijection has recently been generalized to tangled diagrams [28]. Now, a *k*-noncrossing diagram is a special type of *k*-noncrossing tangle and the relevance of Chen's result lies in the fact that the walks in question can be enumerated via the reflection principle. In fact, the reflection principle facilitated the computation of the generating function of *k*-noncrossing canonical pseudoknot RNA [19,26,29]. Subsequent singularity analysis [26,29], showed, that the exponential growth rates of canonical pseudoknot RNA structures are surprisingly small, see Table 1, [26]. For instance, the number of 3-noncrossing, 3-canonical RNA structures with arc-length greater than or equal to four is asymptotically given by

**Table 1:** Exponential growth rates of ⟨*k*, *τ*⟩-structures. We have *k*-noncrossing structures with minimum stack-length greater than or equal to three.

*k*	3	4	5	6	7	8	9
*τ *= 3	2.0348	2.2644	2.4432	2.5932	2.7243	2.8414	2.9480
*τ *= 4	1.7898	1.9370	2.0488	2.1407	2.2198	2.2896	2.3523
*τ *= 5	1.6465	1.7532	1.8330	1.8979	1.9532	2.0016	2.0449
*τ *= 6	1.5515	1.6345	1.6960	1.7457	1.7877	1.8243	1.8569
*τ *= 7	1.4834	1.5510	1.6008	1.6408	1.6745	1.7038	1.7297
*τ *= 8	1.4319	1.4888	1.5305	1.5639	1.5919	1.6162	1.6376
*τ *= 9	1.3915	1.4405	1.4763	1.5049	1.5288	1.5494	1.5677

*cn*^-5 ^2.0348^*n*^,

where *c *is some (explicitly known) constant. This exponential growth rate is very close to Schuster *et al*.'s finding [30] for 2-canonical RNA secondary structures with arc-length greater than or equal to four

(1) 1.4848 *n*^-3/2 ^1.8444^*n*^.

For the analysis presented here, we use the algorithm cross [28], which produces a transparent output. This algorithm does not follow the DP paradigm and generates the mfe-*k*-noncrossing *τ*-canonical structure via a combination of branch and bound, as well as DP techniques. cross inductively constructs *k*-noncrossing, *τ*-canonical RNA structures via motifs. Currently full loop-based energy models are derived an implemented for *k *= 3 and *τ *≥ 3.

Therefore, cross finds the mfe-RNA pseudoknot structure in which there are at most two *mutually *crossing arcs, which has minimum arc-length four and in which each stack has size at least three. While cross is an exponential time algorithm it allows to fold sequences of length 100 with an average folding time of 4.5 minutes.

## Methods

While it is beyond the scope of this paper to present the algorithm cross in detail, the objective of this section is first to sketch its key organization and second to discuss some basic properties of RNA pseudoknot structures. These combinatorial properties enable us to assign a unique, loop-based energy. In the course of our analysis we show that an RNA pseudoknot structure can be constructed via simpler substructures. These serve as the building blocks via which cross derives the mfe-pseudoknot structure. At present time we do not have an algorithm computing the partition function version of cross. For RNA secondary structures, the partition function was obtained 1990 [31], three decades after the first mfe-folding algorithms were derived [32-34]. The partition function is based on a fixed sequence and contains vital statistical information on the probabilities of specific structural configurations of the latter. For any inductively constructed structure class, it allows to compute the base pairing probabilities. In analogy to similar studies in the case of RNA secondary structures [17,35-45], the partition function is for the type of analysis presented here not of key importance. We shall derive statistical information on the sequence-structure relation by mfe-folding a large number of sequences instead of considering the ensemble of structural configurations of a *single *sequence.

### Cross

**Figure 5:**
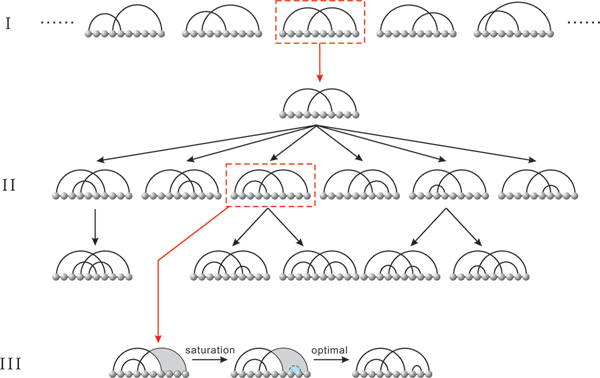
**An outline of cross**. The figure visualizes the three main phases of cross: the generation of motifs (I), the construction of skeleta-trees, rooted in irreducible shadows (II) and the saturation (III), during which, via DP-routines, optimal fillings of skeleta-intervals are derived.

The algorithm cross has three distinct phases: the motif-, skeleton- and saturation-phase, see Figure 5 for an overview. We will here briefly discuss these three parts.

Let ≺ denote the following partial order over arcs

(2) (*i*_1_, *j*_1_) ≺ (*i*_2_, *j*_2_) ⇔ *i*_2 _<*i*_1 _∧ *j*_1 _<*j*_2_,

i.e. an arc *α*_1 _is smaller then *α*_2 _if it is nested in it.

#### I Motifs

**Figure 6:**
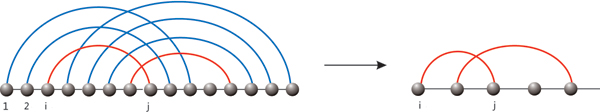
**Cores will in general have 2-arcs**. The structure *δ *(lhs) is mapped into its core *c*(*δ*) (rhs). Clearly d has arc-length ≥ 4 and as a consequence of the collapse of the stack ((1, *j *+ 2), (2, *j *+ 1), (*i*, *j*)) into the arc (i, j), *c*(*δ*) contains the arc (*i*, *j*), which is, after relabeling, a 2-arc.

Let us begin by defining core-structures. A *k*-noncrossing core [29] is a *k*-noncrossing diagram in which all stacks have size one. The core of a structure is obtained by identifying all its stacks by single arcs, keeping the unpaired nucleotides and finally relabeling, see Figure 6.

A ⟨*k*, *τ*⟩-motif is a ⟨*k*, *τ*⟩-diagram over [*n*], having the following properties

(M1) it has a nonnesting core

(M2) all its arcs are contained in stacks of length exactly *τ *= 3 and length *λ *= 4.

**Figure 7:**
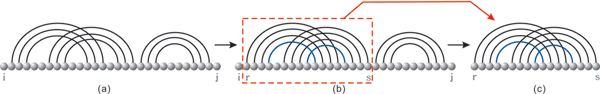
**Motifs, shadows and irreducible shadows**. We display a ⟨3, 3⟩-motif (a) and one of its induced shadows (b), Furthermore we show an irreducible shadow (c), which corresponds to the (b)-substructure contained in the dashed box.

A *m*-shadow is a *k*-noncrossing diagram obtained by successively increasing the stacks of *m *from top to bottom, see Figure 7.

The key observation about motifs is that they can, despite the fact that they exhibit cross-serial dependencies, be generated inductively [46].

#### II Skeleta

Skeleta represent the non-inductive "frames" of pseudoknot RNA, i.e. skeleta entail exactly the cross-serial dependencies, that need to be considered exhaustively. A skeleton, S, is a 3-noncrossing structure, whose core has a connected L-graph. An L-graph is a diagram whose arcs are the vertices and two being adjacent if their corresponding arcs cross [46]. An irreducible shadow, *IS*_*i*,*j*_, over [*i*, *j*]. *IS*_*i*,*j *_is a skeleton which has no nested arcs, see Figure 7. Phase II consists in the generation of all skeleta-trees, which are rooted in irreducible shadows.

#### III Saturation

Given a skeleton, cross saturates or "fills" via context-sensitive DP routines the skeleton-intervals. Note that, while the inserted substructures cannot cross any arc of the skeleton, they will in general contain crossing arcs within themselves.

To summarize, first cross inductively constructs all roots of the skeleta-trees, second cross generates the skeleta-trees themselves and third it saturates the skeleta.

### Loops

We next discuss loops of 3-noncrossing RNA structures. Loops are not only the basic building blocks for the mfe-evaluation but also of importance for the coarse grained notion of pseudoknot-shapes, discussed in Subsection. Let *α *be an arc in the 3-noncrossing RNA structure, *S *and denote by *A*_*S*_(*β*) the set of *S*-arcs that cross *β*. Clearly, we have *β *∈ *A*_*S*_(*α*) if and only if *α *∈ *A*_*S*_(*β*). An arc *α *∈ *A*_*S*_(*β*) is called a minimal, *β*-crossing arc if there exists no *α' *∈ *A*_*S*_(*β*) such that *α' *≺ *α*.

Let the interval [*i*, *j*] denote the sequence

(*i*, *i *+ 1, ..., *j *- 1, *j*).

It is shown in [46] that any 3-noncrossing RNA structure can be uniquely decomposed into the following four loop-types:

**(1) **a *hairpin*-loop is a pair

((*i*, *j*), [*i *+ 1, *j *- 1])

where (*i*, *j*) is an arc.

**(2) **an *interior*-loop is a sequence

((*i*_1_, *j*_1_), [*i*_1 _+ 1, *i*_2 _- 1], (*i*_2_, *j*_2_), [*j*_2 _+ 1, *j*_1 _- 1]),

where (*i*_2_, *j*_2_) is nested in (*i*_1_, *j*_1_).

**Figure 8:**
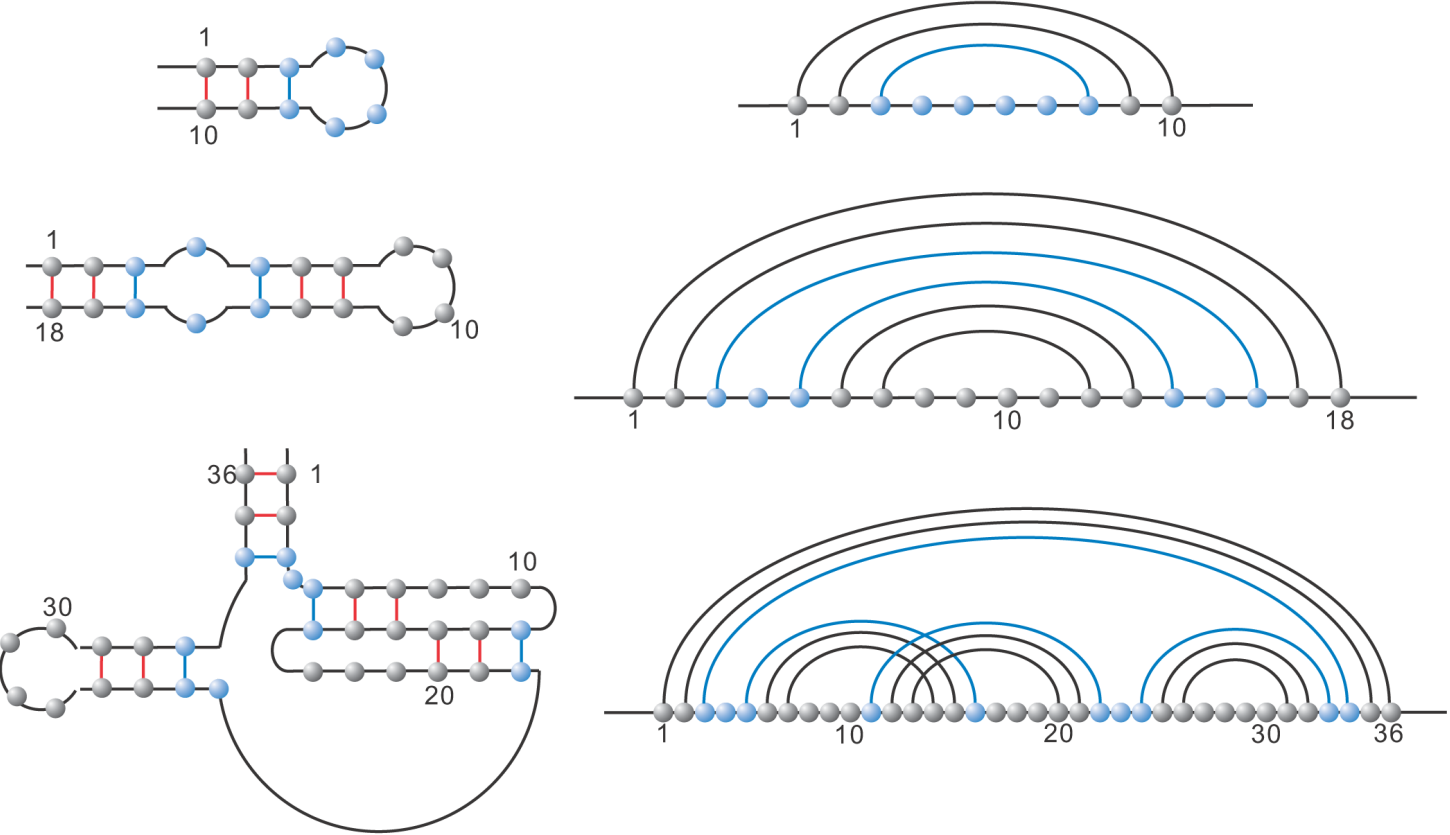
**The standard loop-types **hairpin-loop (top), interior-loop (middle), multi-loop (lower). The lighter base pairs and lighter unpaired bases represent the particular loop.

**(3) **a *multi*-loop, see Figure 8, is a sequence

$$\left((i_{1},j_{1}),[i_{1}+1,\omega_{1}-1],S_{\omega_{1}}^{\tau_{1}},[\tau_{1}+1,\omega_{2}-1],S_{\omega_{2}}^{\tau_{2}},...\right)$$

where $S_{\omega_{h}}^{\tau_{h}}$ denotes a pseudoknot structure over [*ω*_*h*_, *τ*_*h*_] (i.e. nested in (*i*_1_, *j*_1_)) and subject to the following condition: if all $S_{\omega_{h}}^{\tau_{h}}$ = (*ω*_*h*_, *τ*_*h*_), i.e. all substructures are simply arcs, for all *h*, then *h *= 2.

**Figure 9:**
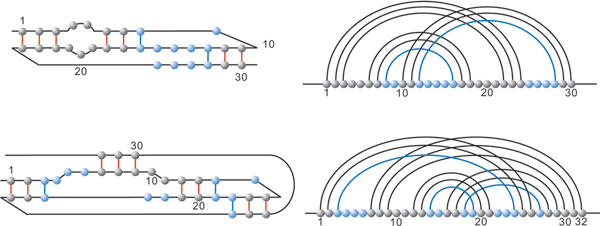
**Pseudoknot-loops**. The lighter base pairs and lighter unpaired bases represent the particular loop. In the lower structure the arc (3, 24) is lighter, since it is minimal crossing with respect to the arc (9, 30), *not *contained in any pseudoknot-loop.

**(4) **a *pseudoknot*, see Figure 9, consisting of the following data:

(P1) a set of arcs

*P *= {(*i*_1_, *j*_1_), (*i*_2_, *j*_2_), ..., (*i*_*t*_, *j*_*t*_)},

where *i*_1 _= min{*i*_*s*_} and *j*_*t *_= max{*j*_*s*_}, such that

(i) the diagram induced by the arc-set *P *is irreducible, i.e. the line-graph of *P *is connected and

(ii) for each (*i*_*s*_, *j*_*s*_) ∈ *P *there exists some arc *β *(not necessarily contained in *P*) such that (*i*_*s*_, *j*_*s*_) is minimal *β*-crossing.

(P2) all vertices *i*_1 _<*r *<*j*_*t*_, not contained in hairpin, interior- or multi-loops.

### Decomposition

We now show that each 3-noncrossing RNA structure can uniquely be constructed by simpler substructures [46]. Furthermore, each 3-noncrossing RNA structure has a unique loop decomposition-the basis of our energy evaluation. We remark that assertion (b) of the following result remains valid for arbitrary crossing number, *k*.

**Theorem**. *Suppose k *≥ 2,*τ *≥ 3.

(a) *Any k-noncrossing, t-canonical RNA structure corresponds to an unique sequence of shadows*.

(b) *Any *⟨3. *τ*⟩-*structure has an unique loop-decomposition*.

**Figure 10:**
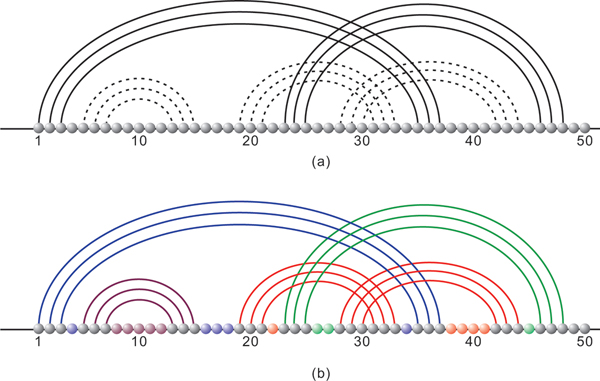
**Shadow and loop-decomposition**. A pseudoknot structure decomposed in its two shadows (top) and its loop-decomposition (bottom).

In Figure 10 we illustrate how these decompositions work.

## Results and discussion

Our results are organized in two sections. First we describe our findings with respect to the statistics of pseudoknot RNA structures and second we present our data with respect to the particular organization of the sequences in neutral networks.

### Minimum free energy RNA pseudoknot structures

In this section we present some key statistics on pseudoknotted RNA structures. In order to put our findings into context we consider two variants of cross: first, cross_3_, which generates 3-noncrossing, 3-canonical mfe-structures and second, cross_4_, which produces 3-noncrossing, 4-canonical mfe-structures.

#### The fraction of pseudoknots

**Figure 11:**
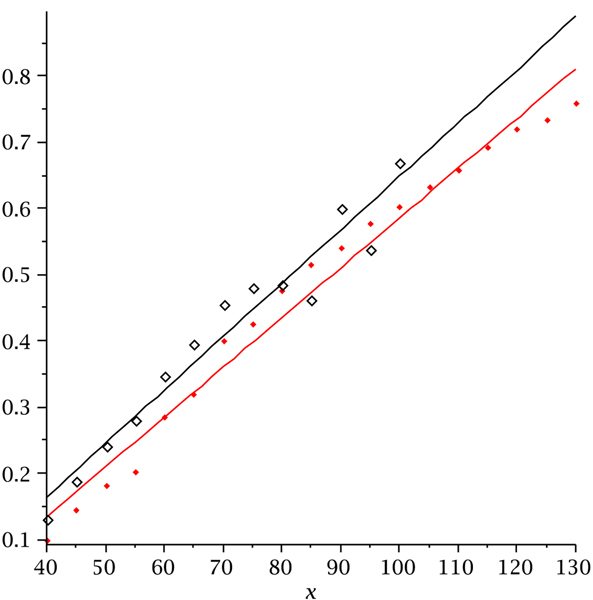
**The fraction of pseudoknot structures over sequence length**. Pseudoknot fractions for cross_3 _(hollow diamonds) and pseudoknot fractions for cross_4 _(solid diamonds).

We next compute the fraction of RNA structures with pseudoknots within all structures for cross_3 _and cross_4_. Figure 11 displays the fraction of structures with pseudoknots as a function of sequence length. It is evident that the fraction of pseudoknotted structures is monotone with respect to the sequence length. Our data are based on folding 2000 random sequences via cross and suggest an linear relation. In particular, for *n *= 100, approximately 50% of the structures folded by both versions of cross contain pseudoknots.

#### Pseudoknot-shapes

**Figure 12:**
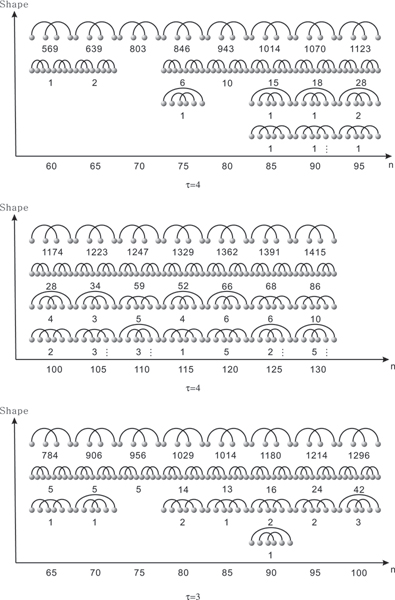
**The dominant pseudoknot shapes for *τ *= 4 and *τ *= 3**. The four dominant shapes displayed from top to bottom according to their frequency. The shapes are obtained sampling 2000 random sequences and labelled by the frequency of their occurrence. For *n *= 40, 45, 50, 55 we only have one shape with frequencies (200, 292, 364, 506), (260, 377, 482, 563), where the coordinates represent cross_4 _and cross_3_, respectively. We have only one shape for *n *= 60, cross_3 _with frequency 697.

**Figure 13:**
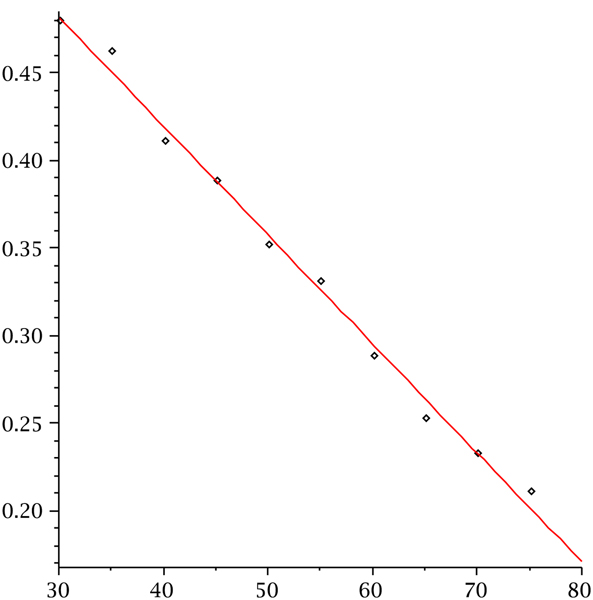
**Fraction of same structures over sequence length**. The fraction of sequences for which cross_4_, generating pseudoknot structures with minimum stack-length four. The data are derived by folding 20, 000 random sequences.

Next we study the dominant pseudoknot-shapes as a function of sequence length. Our notion of pseudoknot-shape is based on *k*-noncrossing cores [29] discussed in Subsection. The shape of a structure *S*, is a subset of the core-arcs, induced by all arcs either contained in pseudoknots or arcs contained in multi-loops which contain nested pseudoknots. In other words, a pseudoknot-shape contains all pseudoknot-arcs and all arcs affecting the energy of pseudoknots, see Figure 12. In Figure 12 we display for cross_3 _and cross_4 _the dominant types. The shape data are obtained by folding 2000 random sequences. In Figure 13 we display the fraction of sequences on which cross_3 _and cross_4 _coincide, based on folding 2000 random sequences.

#### Stack-statistics in pseudoknot RNA

It is wellknown that large stacks contribute to a low mfe of a structure. In this section we relate the distribution of stacks in random structures to the distribution of stacks in mfe-pseudoknot structures generated by cross. This provides insight in what particular spectrum of pseudoknot structures cross produces.

**Figure 14:**
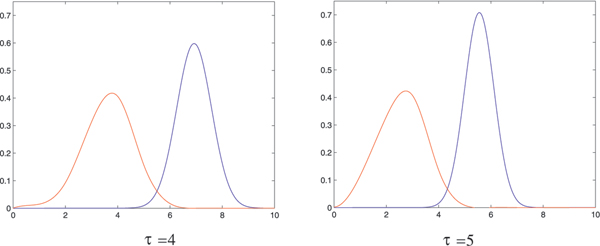
**Stack (loop) numbers in pseudoknot RNA**. We present the stack distributions based on 3000 random sequences of length 80 for cross_4 _(lhs) and cross_5 _(rhs). In addition we display the normal distributions (lighter) as implied by theory with the parameters *μ*_4 _= 0.086554, $\sigma_{4}^{2}$ = 0.0055685 and *μ*_5 _= 0.069467, $\sigma_{5}^{2}$ = 0.0039688.

Let us first discuss the distribution of stacks in random pseudoknot structures. The naive approach would be to generate a random structure and count the number of stacks. However, it is at present time not known how to construct a random pseudoknot structure with uniform probability. Therefore we have to employ a different strategy in order to obtain this distribution for random structures. The key idea [47] is to consider the bivariate generating function

(3) $$T_{k,\tau }(x,u)=\sum_{n\geq 0}\sum_{0\leq t\leq \frac{n}{2}}{\text{T}}_{k,\tau }(n,t)u^{t}x^{n}$$

where T_*k*, *τ*_(*n*, *t*) denotes the number of *k*-noncrossing, *τ*-canonical pseudoknot structures having exactly *t *stacks. **T**_*k*, *τ*_(*x*, *u*) can be computed using the cores introduced in Section. The stack-distribution is now given by

(4) $$\text{P}(X_{k,\tau }^{n}=t)={\text{T}}_{k,\tau }(n,t)/{\text{T}}_{k,\tau }(n)$$

and via singularity analysis one can show that this distribution becomes asymptotically normal with mean *μ*_*k*, *τ *_and variance $\sigma_{k,\tau }^{2}$ given by

(5) $$\mu_{k,\tau }=-\frac{{\gamma^{\prime }}_{k,\tau }(0)}{\gamma_{k,\tau }(0)}$$

(6) $$\sigma_{k,\tau }^{2}={\left(\frac{{\gamma^{\prime }}_{k,\tau }(0)}{\gamma_{k,\tau }(0)}\right)}^{2}-\frac{{\gamma^{\prime \prime }}_{k,\tau }(0)}{\gamma_{k,\tau }(0)}.$$

where *γ*_*k*,*t *_(*u*) is the unique dominant singularity parameterized by *u *= *e*^*s*^. In Table 2 we display the values *μ*_*k*, *τ *_and $\sigma_{k,\tau }^{2}$ for *k *= 2, 3, 4 and *τ *= 3, ..., 7. Accordingly the number of stacks scales linearly with sequence length and so does the number of loops, since each loop corresponds to a stack. In Figure 14 we present the stack distributions of 3000 structures of random sequences folded by cross_4 _and the normal distribution obtained from Table 2 (lhs). Analogously we present the stack distributions of 3000 structures of random sequences folded by cross5 and the normal distribution obtained from Table 2 (rhs).

**Table 2:** Mean and variances. Mean and variances of the normal limit distributions of the numbers of stacks in pseudoknot RNA structures for different *k *and *τ*. We list mean (*μ*) and variance (*σ*^2^).

	*k *= 2		*k *= 3		*k *= 4	
	*μ*	*σ*^2^	*μ*	*σ*^2^	*μ*	*σ*^2^

*τ *= 3	0.090323	0.0189975	0.115473	0.0086760	0.123509	0.0076977
*τ *= 4	0.071677	0.0131316	0.086554	0.0055685	0.091737	0.0049917
*τ *= 5	0.059591	0.0098165	0.069467	0.0039688	0.073166	0.0035769
*τ *= 6	0.051092	0.0077233	0.058149	0.0026885	0.060964	0.0027313
*τ *= 7	0.044774	0.0062991	0.050083	0.0017584	0.052319	0.0021788

### Neutrality and local connectivity

The mapping from sequence to structures plays an important role for evolution [23,43,48]. One of its key roles is to facilitate the search of a sequence-population for better adapted shapes. In tis context, Table 1 contains some nontrivial information about the mapping from RNA sequences into their pseudoknot structures. To be precise, Table 1, in combination with central limit theorems for the number of arcs in *k*-noncrossing RNA structures [49,50] allows us to conclude that there exist exponentially *many k*-noncrossing canonical structures with exponentially large preimages. Indeed, according to Table 1 the exponential growth rate of the number of *k*-noncrossing canonical structures, 3 = *k *= 9 is strictly smaller than four-the growth rate of the space of all sequences over the natural alphabet.

The central limit theorems for the number of arcs of *k*-noncrossing, canonical pseudoknot structures [50] exhibit a mean of 0.39 *n *and a variance of 0.041 *n*. We conclude from this that sequence to structure maps in pseudoknot RNA structures cannot be trivial, since the preimages of particular structures have exponential growth rates strictly smaller than four. As a result the number of canonical pseudoknot structures grows exponentially. Accordingly, a sequence to structure map in pseudoknot RNA necessarily generates exponentially many canonical structures.

In light of this, the interesting question then becomes how the set of sequences folding into a given structure is "organized" in sequence space. The analysis presented in this section is analogous to the investigations for RNA secondary structures [23,51] and can be viewed as a basic protocol for the local statistics of a genotype-phenotype map. The only exception is Subsection, which elaborates on the novel concept of local connectivity [48].

It is only possible to derive local statistics, since, for instance, exhaustive computations of the set of all sequences over the natural alphabet with fixed pseudoknot structure for *n *> 40 is at present time impossible.

#### Neutral walks

Let us consider a fixed RNA structure, *S*. Let furthermore *C*[*S*] denote the set of *S*-compatible sequences, consisting of all sequences that have at any two paired positions one of the 6 nucleotide pairs

(**A**, **U**), (**U**, **A**), (**G**, **U**), (**U**, **G**), (**G**, **C**), (**C**, **G**).

**Figure 15:**
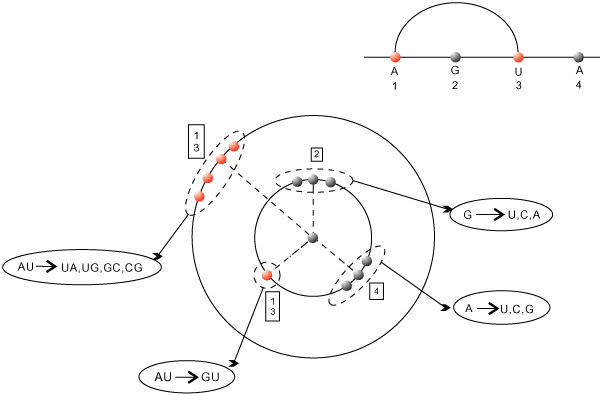
**Compatible neighbors in sequence space**. Diagram representation of an RNA structure (upper right) and its induced compatible neighbors in sequence space (lower left). Note that each base pair gives rise to 5 compatible neighbors exactly one of which is in Hamming distance one.

The structure *S *motivates to consider a new adjacency relation within *C *[*S*]. Indeed, we may reorganize a sequence (*x*_1_, ..., *x*_*n*_) into the pair

(7) $$((u_{1},...,u_{n_{u}}),(p_{1},...,p_{n_{p}})),$$

where the *u*_*j *_denote the unpaired nucleotides and the *p*_*j *_= (*x*_*i*_, *x*_*k*_) all base pairs, respectively, see Figure 15. We can then view *v*_*u *_= (*u*_1_, ..., $u_{n_{u}}$) and *v*_*p *_= (*p*_1_, ..., $p_{n_{p}}$) as elements of the formal cubes $Q_{4}^{n_{u}}$ and $Q_{6}^{n_{p}}$, implying the new adjacency relation for elements of *C *[*S*].

Accordingly, there are two types of compatible neighbors in sequence space: u- and p-neighbors: a u-neighbor has Hamming distance one and differs exactly by a point mutation at an unpaired position. Analogously a p-neighbor differs by a compatible base pair-mutation, see Figure 15. Note however, that a p-neighbor has either Hamming distance one ((**G**, **C**) ↦ (**G**, **U**))) or Hamming distance two ((**G**, **C**) ↦ (**C**, **G**))). We call a u- or a p-neighbor, *y*, a compatible neighbor. If *y *is contained in the neutral network we refer to *y *as a neutral neighbor. This gives rise to consider the compatible- and neutral distance, denoted by *C*(*v*, *v'*) and *N*(*v*, *v'*). These are the minimum length of a *C*[*S*]-path and path in the neutral network between *v *and *v'*, respectively.

**Figure 16:**
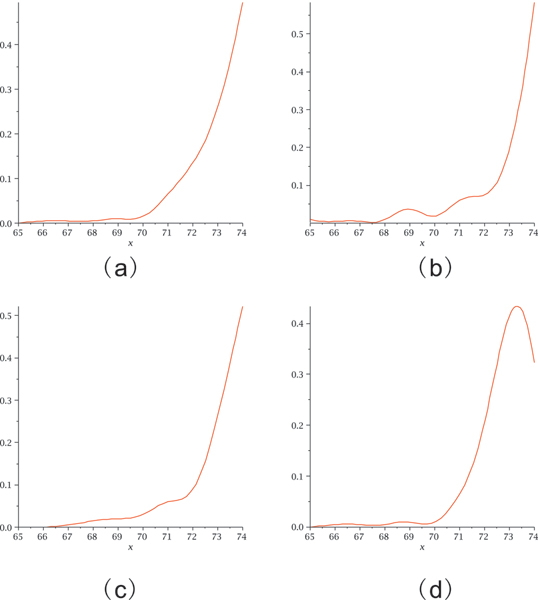
**Neutral walks**. Distance distribution of neutral walks for the corresponding four structures of cross_4 _in Figure 17 based on 200 random paths for each.

**Figure 17:**
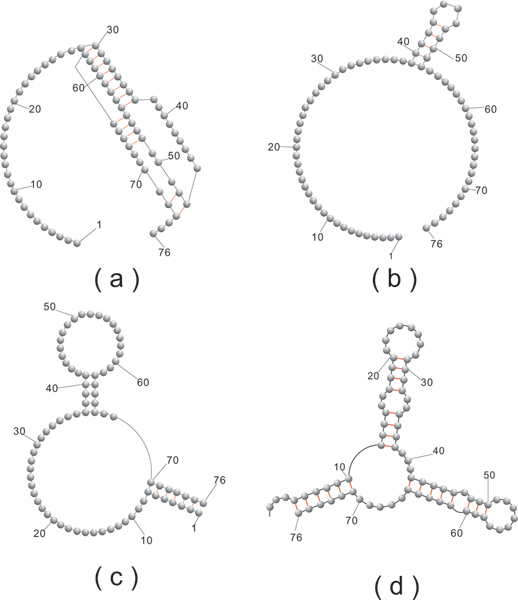
**Four particular pseudoknot structures**. (a).H-loop (b).Hairpin-loop (c).interior-loop (d).tRNA.

Our basic experiment is as follows: We select a (random) sequence, v and fold it into the structure *S*(*v*). We then proceed inductively: assume *v*_*i *_is constructed. We randomly select some neutral (compatible) neighbor of *v*_*i*_, denoted by *v*_*i*+1_, subject to the condition *d*_*H*_(*v*, *v*_*i*+1_) > *d*_*H*_(*v*, *v*_*i*_), where *d*_*H*_(*x*, *y*) denotes the Hamming distance. If no such neighbor exists we choose some *v*_*i*+1 _≠ *v*_*i *_with the property *d*_*H*_(*v*, *v*_*i*+1_) = *d*_*H*_(*v*, *v*_*i*_). If all neutral *v*_*i*_-neighbors satisfy *d*_*H*_(*v*, *v*_*i*+1_) <*d*_*H*_(*v*, *v*_*i*_) we stop and output the integer *d*_*H*_(*v*, *v*_*i*_). In Figure 16 we study 200 neutral walks for the following four structures: first an *H*-pseudoknot loop structure (a), second a hairpin-loop structure (b), third an interior-loop structure (c) and finally the phenylalanine tRNA structure (d), see Figure 17. Our findings are in accordance with those for RNA secondary structures. One can easily neutrally traverse sequence space, suggesting the picture of vast, connected networks composed by neutral sequences.

#### Neutral neighbors

**Figure 18:**
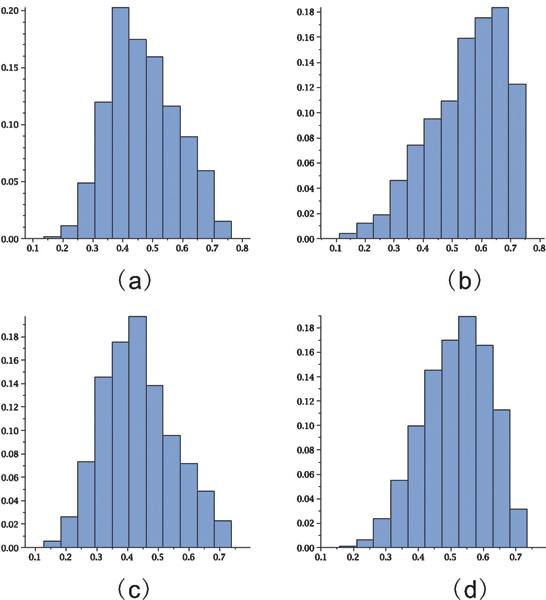
**The fraction of neutral neighbors for the pseudoknot structures**. (a), (b), (c) and (d) are based on sequences in their random 200 paths for cross_4_.

**Figure 19:**
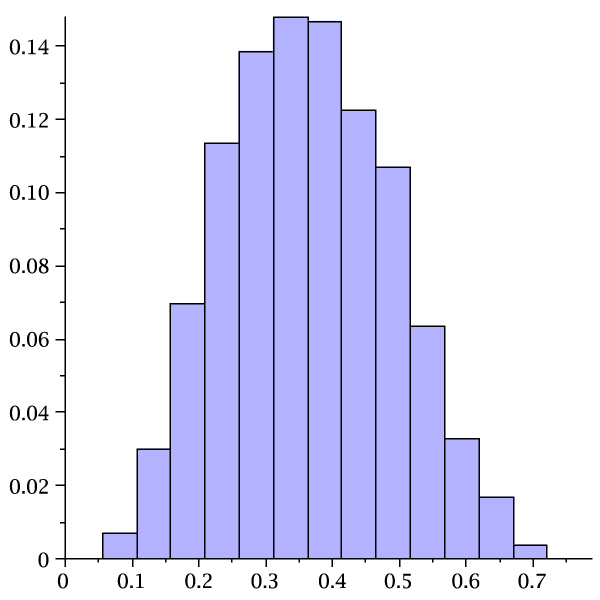
**Neutral fraction distribution**. Neutral fraction distribution of 20, 000 random sequences for cross_4_.

Complementing the analysis of neutral walks, we study now the distribution of neutral neighbors. Recall that a neutral neighbor of a sequence *v *with respect to the structure *S *= *S*(*v*) is a u- or a p-neighbor, *y*, contained in the neutral network of *S*. It has Hamming distance one or two, depending on whether it is induced by a point or base pair mutation, see Figure 15. The distribution of neutral neighbors provides relevant information about the mutational robustness of the structure *S*. The data presented here, are obtained in the course of the neutral walk experiments, displayed in Figure 16. They are given in Figure 18. In order to put things into context we also present in Figure 19 the distribution of neutral neighbors for 10000 random sequences folded by cross_4_.

#### Local connectivity

Connectivity of a subgraph, Γ_*n*_, of an *n*-cube alone does not imply that a small Hamming distance implies a small distance in Γ_*n*_. For neutral sequences this means that two neutral sequences with Hamming distance less than four, are possibly connected via a neutral path of much greater length. Evidently, for molecular evolution it is therefore not connectivity but the existence of these short paths what matters. Local connectivity is a property which guarantees the existence of these short paths. If Γ_*n *_is locally connected then a small Hamming distance does imply a Γ_*n*_-distance scaled by at most a factor of Δ > 0. We shall begin by studying local connectivity for random induced subgraphs of *n*-cubes, i.e. where we select sequences with independent probability *λ*_*n*_. Then we transfer the derived concepts to neutral networks of RNA pseudoknot structures.

We call Γ_*n *_is locally connected if and only if almost surely (a.s.)

$$\begin{array}{cc} \left(†\right) & \begin{array}{cc} \exists \Delta >0; & d_{\Gamma_{n}}(v,v^{\prime })\leq \Delta d_{Q_{2}^{n}}(v,v^{\prime }) \end{array} \end{array},$$

provided *v*, *v' *are in Γ_*n*_. We immediately observe that, trivially, for any *finite n *such Δ exists. However, the key point is that (†) employs the notion "almost surely", i.e. it holds for arbitrary *n*.

Random graph theory [48] shows that on the one hand, for *λ*_*n *_smaller than *n*^*δ*^/$\sqrt{n}$, where *δ *> 0 is arbitrarily small, there exists a.s. no finite Δ satisfying (†). On the other hand, for *λ*_*n *_larger than or equal to *n*^*δ*^/$\sqrt{n}$, there exists a.s. some finite Δ satisfying (†). In other words, there exists a threshold value for local connectivity. Since random subgraphs of *n*-cubes have giant components for *λ*_*n *_= (1 + *ε*)/*n*, where *ε *> 0 [52] we can conclude that local connectivity emerges distinctly later in the evolution of random subgraphs of *n*-cubes.

**Figure 20:**
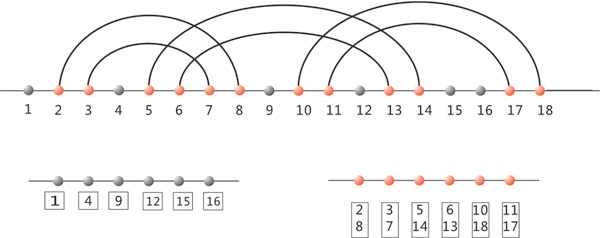
**Deriving the two subcubes $Q_{4}^{n_{u}}$ and $Q_{6}^{n_{p}}$**. A structure gives rise to rearrange its compatible sequences into unpaired and paired segment. The former is a sequence over the original alphabet **A**, **U**, **G**, **C **and for the latter we derive a sequence over the alphabet of base pairs, (**A**, **U**), (**U**, **A**), (**G**, **U**), (**U**, **G**), (**G**, **C**), (**C**, **G**)

Suppose we are given a structure *S *and sequence *v*, contained in its neutral network. By construction, local connectivity refers to the two *n*-cubes $Q_{4}^{n_{u}}$ and $Q_{6}^{n_{p}}$ induced by *S*, see Figure 20. Let

*C*_2 _= |{*v'*| *C*(*v*, *v'*) = 2}|

be the cardinality of the set of sequences in compatible distance two. Then the degree of local connectivity of *S *at *v *is given by

(8) $$\begin{array}{cc} D_{S}(v)=|\{v^{\prime }|C(v,v^{\prime })=2, & N(v,v^{\prime })=4\}|C_{2}^{-1} \end{array}.$$

In other words, *D*_*S*_(*v*) is the fraction of locally connected vertices of the compatible distance two neighbors of *v*, that can be obtained via a neutral path of length at most four.

**Figure 21:**
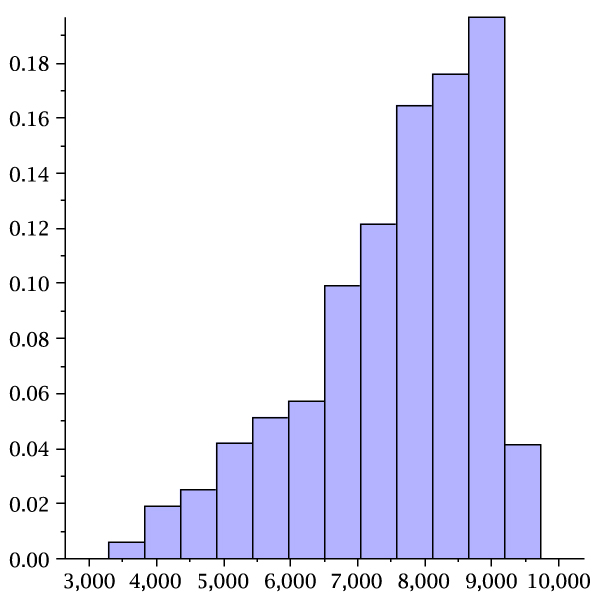
**Local connectivity of the UTR-pseudoknot**. Presented are the numbers of locally connected sequences during various neutral walks. The degree of local connectivity, *D*_*S*_(*v*), is one for all steps.

We perform the following experiment: we consider neutral walks for the UTR-pseudoknot structure of the mouse hepatitis virus displayed in Figure 1, see Subsection. Along these walks we compute the locality degree *D*_*S*_(*v*_*i*_) and the total number of locally connected sequences. Our findings are presented in Figure 21. We can report that the degree of local connectivity is, as suggested by random graph theory, almost 100%.

## Conclusion

RNA pseudoknot structures-in particular their statistical properties-are a fascinating and new territory. To our knowledge the only statistical data beyond RNA secondary structures were derived for bi-secondary structures in [53,54]. The structural concept of *k*-noncrossing canonical RNA structures and the resulting sequence to structure map employed for our experiments is new and represents a natural generalization of RNA secondary and bi-secondary structures. To be precise, bi-secondary structures are exactly planar 3-noncrossing RNA structures [19].

It is clear, that for sequence-length less than or equal to 100 we only encounter pseudoknots of limited complexity. Our findings presented in Figure 12 provide a transparent picture of which pseudoknot-shapes dominate for given sequence length. These results, in combination with the data on the fractions of pseudoknotted structures over sequence length show, that for *n *= 80 we have approximately 35% structures with nontrivial pseudoknots. In addition it is striking that basically all folded structures are irreducible, i.e. only a very small fraction can be decomposed into several independent substructures. This is of interest since decomposable structures can be folded much faster. It is known, [55] that Dyck-paths, i.e. path starting at the origin, having only up (1, 1), or down (1, -1) steps which end on the *x*-axis, decompose on average into three irreducible parts. This is of relevance, since a slight generalization of Dyck-path, the Motzkin-paths, having additional horizontal steps, correspond to secondary structures. Our findings suggest, that while secondary structures, decompose nontrivially, higher and higher crossing numbers change the picture. This complicates the computation of mfe-pseudoknot RNA due to their imminent irreducibility.

Both versions of cross produce analogous findings, confirming the generality of our results. The vast majority of pseudoknot-shapes is of a single type. As expected, cross_3 _exhibits more structural variety due to the fact that its minimum stack-length is only three. The ratio of pseudoknot structures shifts significantly from *n *= 80 to *n *= 100 to approximately 50%. We can conclude from this that pseudoknots cannot be ignored, they evidently become the dominant structure class for *n *greater than or equal to 100. Figure 13 shows that the fraction of sequences for which cross_3 _and cross_4 _coincide, decreases linearly as a function of sequence length. This indicates that larger and larger sequences will exhibit more subtle structural elements whose emergence is facilitated by stabilizing large stacks.

Furthermore, the mfe-pseudoknot structures generated by cross are far from being random. The central limit theorems for random *k*-noncrossing canonical RNA structures, given in Table 2 imply, that stacks and consequently loops scale linearly with the sequence length. Figure 14 clearly shows that the mfe-structures, generated by cross_4 _and cross_5_, have for *n *= 76 two stacks less than random 3-noncrossing structures with minimum stack-length greater than four and five, respectively. This deviation is significant and indicates that mfe-pseudoknot structures are far from "typical" random structures. We remark that, while it is straightforward to generate random RNA secondary structures, it is nontrivial to obtain random pseudoknot structures. In particular, at present time, no polynomial time algorithm is known which generates a random 3-noncrossing RNA structure with uniform probability.

The organization of the sequences contained in neutral networks of RNA pseudoknot structures seems to be very analogous to the neutral networks of RNA secondary structures [23]. Figure 16 shows that neutral walks can effectively traverse sequence space and the fractions of neutral neighbors, presented in Figure 18 and Figure 19 suggest a high degree of neutrality.

We discussed in Subsection local connectivity, a property of neutral networks which implies the existence of short, neutral paths. It is apparent that local connectivity is of central importance for molecular evolution and any type of evolutionary optimization. It has been shown in [48] that local connectivity is a prerequisite for preserving any type of sequence specific information. Having a random graph threshold value localized at 1/$\sqrt{n}$, local connectivity appears much later than connectivity, being localized at 1/*n*. However, the high neutrality degrees of RNA pseudoknot structures of Figure 18 and Figure 19 imply locally connected neutral networks. Our findings for the UTR-pseudoknot structure of the mouse hepatitis virus of length 56, given in Figure 21, confirm the local connectivity of neutral networks of particular pseudoknot RNA structures. At all steps of the neutral walks almost all sequences are locally connected.

## List of abbreviations used

UTR: Untranslated Region; HDV: Hepatitis Delta Virus; DP:dynamic program; lhs: left hand side; rhs: right hand side.

## Competing interests

The authors declare that they have no competing interests.

## Authors' contributions

All authors contributed equally to this paper.
